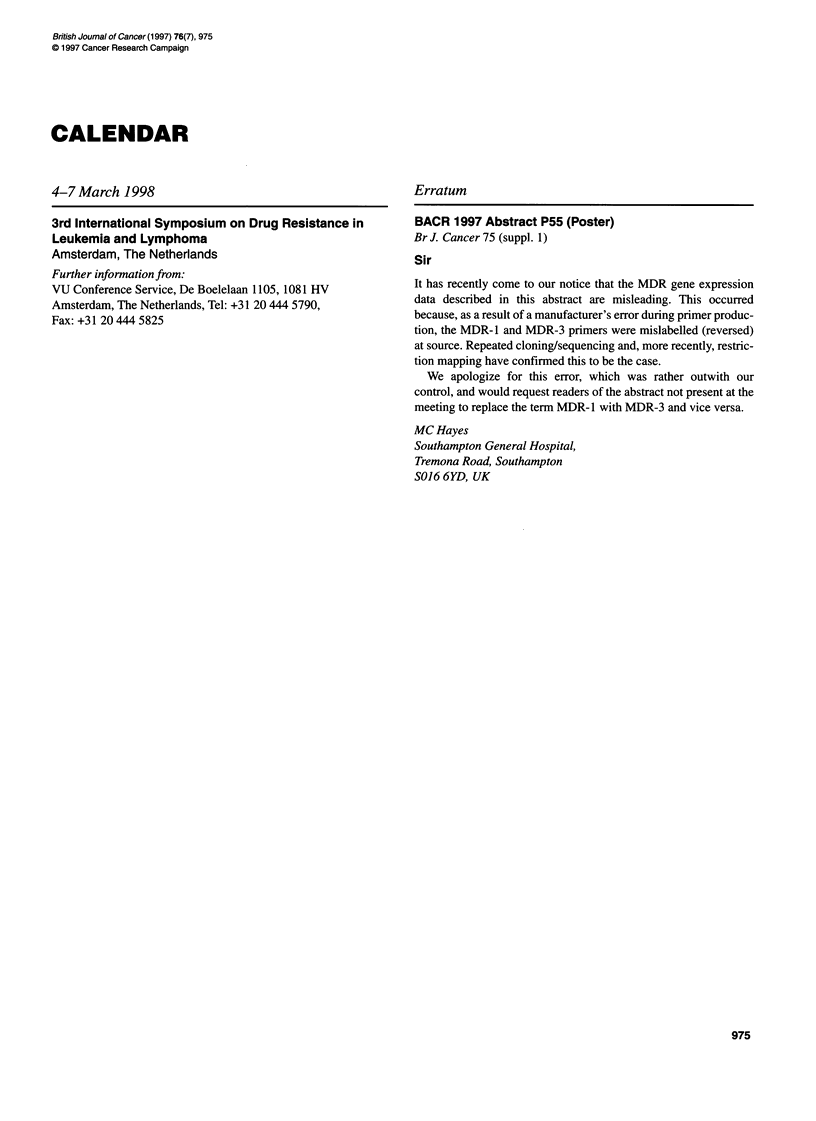# Calendar

**Published:** 1997

**Authors:** 


*British Journal of Cancer* (1997) **76**(7), 975

© 1997 Cancer Research Campaign

### CALENDAR

*4–7 March 1998*

**3rd International Symposium on Drug Resistance in Leukemia and Lymphoma**
Amsterdam, The Netherlands

*Further information from:*
VU Conference Service, De Boelelaan 1105, 1081 HV Amsterdam, The Netherlands, Tel: +31 20 444 5790, Fax: +31 20 444 5825